# The human microbiome in cancer: Not just a sidekick anymore

**DOI:** 10.1590/1678-4685-GMB-2025-0236

**Published:** 2026-05-22

**Authors:** Jennifer Vieira Gomes, Suzana Pinheiro de Oliveira Ribeiro, Gabriella Marins Cassiano do Nascimento, Danielle Oliveira dos Santos, Diego José Gomes de Paula, Sheila Coelho Soares Lima, Tatiana de Almeida Simão

**Affiliations:** 1 Universidade do Estado do Rio de Janeiro (UERJ), Instituto de Biologia Roberto Alcantara Gomes, Departamento de Bioquímica, Rio de Janeiro, RJ, Brazil.; 2 Instituto Nacional de Câncer (INCA), Coordenação de Pesquisa e Inovação, Divisão de Pesquisa Clínica e Desenvolvimento Tecnológico, Rio de Janeiro, RJ, Brazil.; 3 Instituto Nacional de Câncer (INCA), Coordenação de Pesquisa e Inovação, Banco Nacional de Tumores e DNA, Rio de Janeiro, RJ, Brazil.

**Keywords:** Microbiome, dysbiosis, carcinogenesis, tumor microenvironment, therapeutic response

## Abstract

The human microbiome is increasingly recognized as a dynamic element in cancer biology. Studies across breast, prostate, lung, colorectal, and cervical tumors reveal that microbial communities influence carcinogenesis, immune regulation, and treatment outcomes. When the balance of these microorganisms is altered, inflammation becomes chronic, metabolism is disrupted, and signaling pathways such as NF-κB, IL6-STAT3, and β-catenin are activated. Bacterial metabolites and genotoxins, including colibactin and bile acids, may damage DNA and reshape the epigenetic landscape. Distinct microbial profiles have been linked to prognosis and to patient responses to chemotherapy and immunotherapy. The presence of beneficial taxa, such as *Akkermansia muciniphila* and *Ruminococcus*, has been associated with improved response to immune checkpoint inhibitors. At the same time, antibiotic-induced depletion of gut microbiome can reduce therapeutic efficacy. Strategies that help restore microbial balance, including probiotics, dietary interventions, and fecal microbiota transplantation, are being explored as complementary therapies. Although methodological differences and contamination remain challenges, the growing body of evidence indicates that the microbiome is a measurable and modifiable component of tumor ecosystems with strong potential for diagnostic, prognostic, and therapeutic applications in precision oncology.

## Introduction

Despite significant scientific and technological advances over recent decades, cancer continues to be one of the leading health challenges worldwide. Its social and economic impacts vary across cancer types, geographic regions, and sexes (Sung et al., 2021). The disease accounts for roughly one in every six deaths globally and about one in four deaths from chronic noncommunicable diseases (Sung *et al*., 2021; Ferlay *et al*., 2024). According to GLOBOCAN 2022, breast, prostate, lung, colorectal, and cervical cancers are the five most common cancers in the world, evidencing them as public health issues worldwide and reflecting both their biological complexity and the influence of environmental exposures (Ferlay *et al*., 2024). 

**Figure 1 -:**
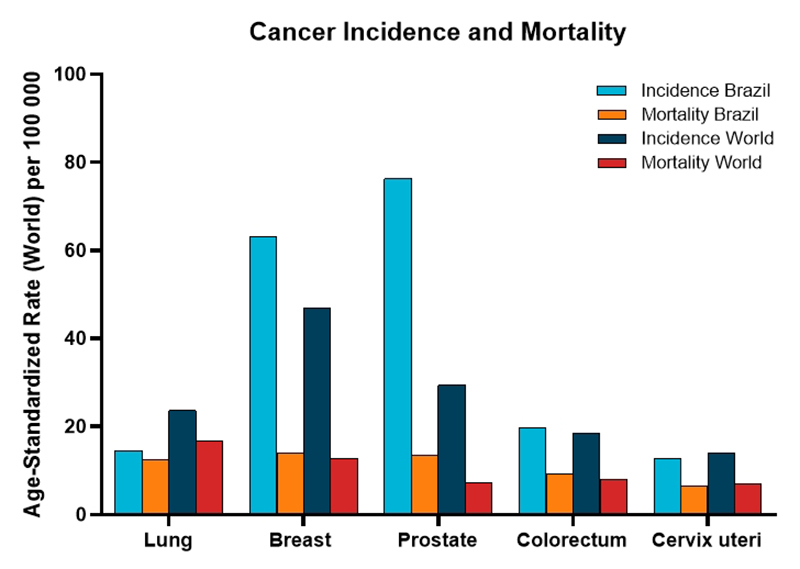
Age-standardized incidence and mortality rates (per 100,000). Global - Incidence: breast 46.8; prostate 29.4; lung 23.6; colorectal 18.4; cervical 14.1. Mortality: lung 16.8; breast 12.7; colorectal 8.1; prostate 7.3; cervical 7.1. Brazil - Incidence: prostate 77.9; breast 66.5; colorectal 21.1; cervical 15.4; lung 15.1. Mortality: breast 13.8; prostate 13.7; lung 12.3; colorectal 9.0; cervical 6.3. Global and national patterns are similar regarding the most frequent cancers but differ in magnitude, reflecting demographic, environmental, and healthcare disparities (created using R software - v. 4.3.2).

In Brazil, excluding nonmelanoma skin cancers, an estimated 483,000 new cancer cases occured annually between 2023 and 2025. Among these cases, over 40% correspond to prostate, breast, lung, and cervical cancers (INCA, 2023). This epidemiological pattern highlights the need for more specific prevention strategies and for the development of diagnostic, prognostic, and therapeutic biomarkers. The global and Brazilian incidence and mortality rates for the five most frequent cancers are summarized in a comparative graphic (Figure 1).

Among alterations that lead to cancer development and/or that are induced by the carcinogenesis process, more recently, polymorphic microbiomes have emerged as a promising hallmark (Hanahan, 2022). But the association between microorganisms and cancer is not new; it has been progressively elucidated over more than a century of research. In 1911, Peyton Rous demonstrated that a transmissible agent could induce tumors in chickens, inaugurating the concept of viral carcinogenesis. Decades later, the discovery of human gammaherpes virus 4 (Epstein-Barr virus) in Burkitt lymphoma and the identification of HPV16 and HPV18 in cervical carcinoma confirmed that persistent infections can actively participate in human carcinogenesis (Epstein *et al*., 1964; Dürst et al., 1983; Boshart et al., 1984). Launched in 2007, the Human Microbiome Project (Human Microbiome Project Consortium, 2012) fundamentally altered our view of the human body’s microbial landscape. It highlighted commensal microorganisms as key players within the tumor microenvironment, modulating immunity, metabolism, and inflammatory pathways. Since then, distinct microbial communities have been identified within breast, prostate, lung, colorectal, and cervical tumors, suggesting species-specific roles in tumor initiation and therapeutic response (Kostic et al., 2012; Cavarretta et al., 2017; Nejman et al., 2020).

**Figure 2 -:**
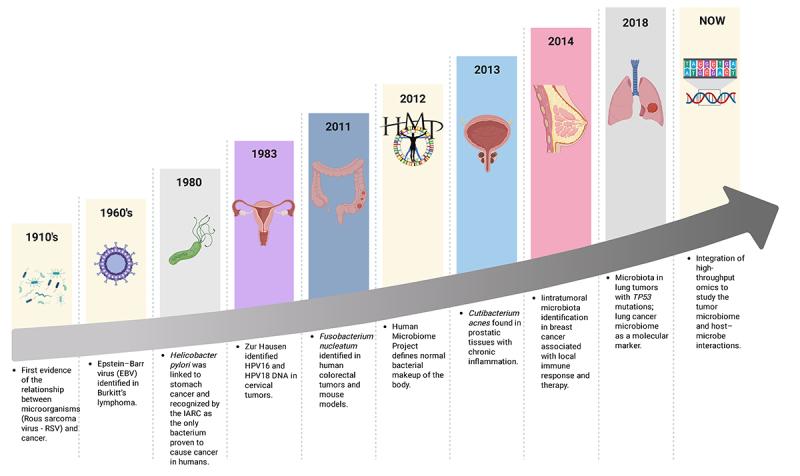
Historical milestones linking microorganisms to cancer development and the emergence of the tumor microbiome field. From the first evidence connecting Rous sarcoma virus (RSV) to cancer in the 1910s to the identification of Epstein-Barr virus (EBV) in Burkitt’s lymphoma and *Helicobacter pylori* in gastric cancer, successive discoveries have revealed the microbial contribution to carcinogenesis. The identification of *Fusobacterium nucleatum* in colorectal tumors (2011) and *Cutibacterium acnes* in prostatic inflammation (2013) expanded this concept beyond viruses. The Human Microbiome Project (2012) defined the baseline composition of the Human microbiome, while subsequent studies identified intratumoral microbiome in breast (2014) and lung (2018) cancers. Currently, high-throughput technologies are enabling integrative analyses of tumor microbiomes and host-microbe interactions across multiple cancer types (created in BioRender. https://BioRender.com/d051x65).

This historical progression is summarized in Figure 2, which highlights key milestones linking microorganisms to human carcinogenesis and the rise of microbiome research. In this context, researchers increasingly recognize the need to move beyond traditional assessments of risk factors and morphology, and to integrate molecular and ecological aspects of cancer. The human microbiome has become one of the most promising areas in this regard, with growing evidence that it can shape tumor development and influence how patients respond to treatment. 

The microbiome was first defined as a microbial community characteristic of a well-defined habitat, encompassing not only the microorganisms themselves but also the physicochemical properties of the environment and their “theatre of activity,” which refers to the network of functional interactions within this ecosystem (Whipps et al., 1988). With the advent of omics technologies, this concept has expanded, and the microbiome is now recognized as a dynamic system comprising microorganisms, their genomes, metabolites, ecological structures, and interactions with the host, playing essential roles in both physiological and pathological processes (Berg et al., 2020).

In this review, the term microbiome is used from an integrative perspective, particularly in the context of microbiota-immune system interactions and cancer. Disruptions in its composition and diversity, known as dysbiosis, have been linked to multiple stages of carcinogenesis, from tumor initiation to metastatic spread and resistance to therapy (Garrett, 2019; Helmink et al., 2019).

Specific microorganisms have long been linked to cancer: *Helicobacter pylori* is causally associated with gastric carcinoma and lymphoma, while *Fusobacterium nucleatum* (*F. nucleatun*) contributes to colorectal tumorigenesis and chemoresistance (Castellarin et al., 2012; Kostic et al., 2012). More recently, microbial signatures have been identified across a wide range of solid tumors, including prostate, breast, and lung cancers, once thought to be sterile or minimally colonized (Nejman et al., 2020; Cullin et al., 2021). These discoveries, enabled by high-throughput sequencing and improved contamination control, have shifted the paradigm of tumor biology to encompass tumor-resident and systemic microbiota as active participants in disease behavior and therapeutic response (Nejman *et al*., 2020; Cullin *et al*., 2021; Liu et al., 2024).

## Methodological approach

This paper presents a narrative review based on a broad, non-systematic survey of the literature on microbiome-cancer interactions, with an emphasis on lung, prostate, colorectal, breast, and cervical tumors. Literature searches were conducted in PubMed, Scopus, and Web of Science using combinations of relevant keywords. Original articles and relevant reviews published in English over the last decade were prioritized, while case reports, conference abstracts, and studies outside the scope of this review were excluded. This review also draws on evidence synthesized in previously published systematic reviews, which are cited where appropriate. Paper selection was guided by relevance to the objectives of the review.

## Breast Cancer

Breast cancer is a biologically heterogeneous disease and is commonly classified into molecular subtypes, including hormone receptor-positive tumors (Luminal A and Luminal B), HER2-positive tumors, and triple-negative breast cancer (TNBC), which differ in prognosis, therapeutic response, and tumor biology. TNBC is consistently associated with poorer clinical outcomes when compared with other molecular subtypes of breast cancer (Perou et al., 2000; Sørlie et al., 2001). TNBC is defined by the absence of estrogen receptor (ER), progesterone receptor (PR), and HER2 expression and is associated with higher histological grade, elevated proliferative index, early recurrence, increased risk of visceral and distant metastases, and poorer overall and disease-free survival compared with other breast cancer subtypes (Dent et al., 2007; Lehmann et al., 2011). 

For many years, the breast was thought to be a sterile organ. However, advances in high-sensitivity sequencing have revealed that even healthy breast tissue contains a small but metabolically active microbial community (Human Microbiome Project Consortium, 2012). These microorganisms are not mere contaminants; both microbial DNA and viable bacteria have been consistently detected in normal parenchyma and tumor samples (Hieken et al., 2016; Urbaniak et al., 2016). In line with these observations, breast tumors exhibit relatively high intratumoral alpha diversity, reflecting microbial richness and evenness, together with increased bacterial abundance (i.e., higher bacterial load) compared with normal-adjacent tissue, which displays intermediate profiles relative to healthy breast tissue, supporting the existence of distinct tumor-associated microbial communities (Nejman et al., 2020). Consistently, beta diversity analyses reveal differences in community structure between healthy breast tissue and cancer-associated (normal-adjacent) samples, whereas tumor and adjacent tissue profiles are broadly similar, indicating that local tumor context shapes breast-associated microbial communities (Urbaniak *et al*., 2016). Several genera recur across studies: *Sphingomonas* has been predominantly observed in healthy breast tissue, while malignant tumors often show enrichment of *Methylobacterium, Escherichia, Fusobacterium* and *Prevotella* (Banerjee et al., 2015; Luo et al., 2025).

One conceptual model linking these findings is the gut-breast axis, which proposes that intestinal microbiota can influence breast tissue homeostasis and carcinogenesis through systemic signaling mechanisms (Kwa et al., 2016; Parida et al., 2021). Microorganisms and their metabolites originating in the gut may influence mammary tissue directly through hematogenous or lymphatic dissemination and indirectly by altering systemic metabolism and immune responses. In women with breast cancer, the gut microbiota shows a distinct composition compared with that of healthy individuals. Notably, an increased abundance of *Bifidobacterium* in circulation may reflect gut microbial translocation. In contrast, a positive association between reduced *Ligilactobacillus* abundance and lower levels of 4-hydroxybenzoic acid suggests the loss of metabolically protective microbial functions that could contribute to systemic dysregulation (Peng et al., 2024).

Experimental work demonstrates that enterotoxigenic *Bacteroides fragilis* (ETBF), a procarcinogenic colon microbe, can promote mammary tumorigenesis and metastatic progression by activating oncogenic pathways, including Notch and β-catenin axes (Parida et al., 2021). These data support the idea that microbial translocation and metabolite-driven systemic signaling converge to shape the breast tumor microenvironment.

Microbial metabolites have been increasingly recognized as potential mediators of host-tumor interactions, although their effects on breast cancer appear to be context-dependent. The bacterial enzyme β-glucuronidase, which deconjugates estrogens in the gut, affects systemic estrogen recirculation and may influence the biology of hormone receptor-positive tumors (Kwa et al., 2016). In addition, genotoxins such as colibactin, produced by Escherichia coli harboring the polyketide synthase island, can cause DNA double-strand breaks and drive genomic instability, while secondary bile acids and other microbial products have been implicated in epigenetic modulation and tumor-promoting inflammation (Nougayrède et al., 2006; Ridlon et al., 2014).

Immune-microbiome crosstalk is increasingly recognized as central in breast tumor biology. Specific microbial signatures have been associated with infiltration of cytotoxic CD8⁺ T cells and activation of proinflammatory pathways in tumor tissue (Hieken et al., 2016; Nejman et al., 2020). Such modulation may influence tumor progression and response to therapy. Hormone receptor-positive breast tumors seem to be particularly influenced by the estrobolome, the gut microbial community that metabolizes estrogens, given its role in regulating systemic estrogen metabolism. In contrast, TNBC frequently present microbiota associated with higher inflammatory activity and immune cell infiltration, features that may influence tumor progression and treatment response (Kwa et al., 2016). Despite significant progress in this area, methodological challenges remain. It is known that breast tissue contains very low levels of microbial biomass; the risk of contamination is high, making it essential to apply strict experimental controls and robust computational analyses to ensure reliable results (Eisenhofer et al., 2019). 

Clinically, the possibility of manipulating the microbiome to improve breast cancer outcomes is emerging. Preclinical and early-phase clinical studies suggest that gut microbial composition can influence the efficacy of immune checkpoint inhibitors (anti-PD-1/PD-L1), chemotherapy, and endocrine therapy (Gopalakrishnan et al., 2018; Routy et al., 2018). Although most robust evidence comes from gastrointestinal and melanoma cohorts, early data in breast cancer also point in this direction: distinct gut and tumor-associated microbiota have been linked to response profiles and immune modulation in triple-negative disease (Luo et al., 2025). *Akkermansia muciniphila* and *Bifidobacterium longum* enrichment has been associated with enhanced immunotherapy response, while dysbiosis correlates with therapeutic resistance (Gopalakrishnan *et al*., 2018; Routy *et al*., 2018; Abdul Nazeer et al., 2025). These findings are beginning to shape breast cancer research and clinical trials evaluating probiotics, prebiotics, and fecal microbiota transplantation as adjuvant interventions (Banerjee et al., 2015; Urbaniak et al., 2016; Abdul Nazeer *et al*., 2025).

## Prostate cancer

Prostate cancer has long been viewed as a disease driven by androgen signaling and somatic mutations. However, it is now recognized that its development occurs within a complex inflammatory and metabolic microenvironment that is, at least in part, shaped by the microbiome (Sfanos and De Marzo, 2012; Lachance et al., 2024). Dysbiosis appears to play an important role in this process, influencing local prostatic inflammation, immune modulation, and even treatment resistance, a scenario that closely resembles what has been described in breast cancer.

Evidence supports both direct and indirect microbial contributions to prostate carcinogenesis. At the tissue level, prostatic infections with uropathogens such as *E. coli* can disrupt epithelial barriers, recruit neutrophils and macrophages, and promote chronic inflammation (Kustrimovic et al., 2023). This leads to the production of reactive oxygen and nitrogen species, which in turn damage DNA, promote mutations, and induce epigenetic changes that favor neoplastic transformation and the development of prostatic intraepithelial neoplasia (Kustrimovic *et al*., 2023). Beyond local infection, growing evidence points to a gut-prostate connection. Microorganisms and their metabolites may reach the prostate through systemic circulation or influence it indirectly by altering immune and metabolic pathways that regulate tissue homeostasis.

Several lines of evidence suggest that a loss of gut microbial diversity may be linked to more aggressive forms of prostate cancer. In experimental models carrying *PTEN* and *Rb1* deletions, mutations frequently observed in advanced and castration-resistant disease, reduced alpha diversity has been associated with faster tumor growth and impaired immune regulation (Lachance et al., 2024). In this context, Lachance *et al*. (2024)reported significant reductions in alpha diversity (8.3 ± 2.6% in patients with high tumor volume and up to 17.9 ± 3.1%; p = 0.02). Beta-diversity analysis showed no separation according to tumor volume in the full cohort (p = 0.48). In contrast, significant compositional shifts were observed in high-PSA patients and in murine tumor models (p = 0.03-0.001) (Lachance *et al*., 2024). 

Clinical data show a similar profile. In advanced stages of prostate cancer, the gut microbiota often shifts, with the phylum *Pseudomonadota* becoming more abundant than beneficial genera such as *Lactobacillus* and *Bifidobacterium,* and with markedly reduced levels of these commensal bacteria (Zhong et al., 2022). Consistently, Zhong and colleagues investigated gut microbiota composition using *16S rRNA* sequencing of murine fecal samples from groups with and without antibiotic exposure. Alpha diversity analyses revealed that microbiota reconstitution in antibiotic-exposed mice led to an increase in alpha diversity compared with antibiotic-treated animals. Beta-diversity analyses indicated similar bacterial community compositions between antibiotic-exposed mice and those receiving microbiota transplantation, as well as between non-antibiotic-exposed controls and their corresponding transplantation group, suggesting no major differences in overall community structure between these paired groups (Zhong *et al*., 2022).

This altered microbial landscape can compromise the intestinal barrier, allowing bacterial products, such as LPS, to leak into the bloodstream and sustain systemic inflammation. Increased levels of LPS have also been found in prostate tumor tissue, where they trigger NF-κB-IL6-STAT3 signaling, driving the expression of genes that foster tumor cell growth and resistance to chemotherapy, including *MYC* and *CCND1*. In preclinical studies, pharmacologic blockade of STAT3, using agents such as Stattic, partially reversed these effects, underscoring the functional link between dysbiosis, inflammation, and tumor progression (Zhong et al., 2022).

Emerging data have identified additional bacterial taxa-among them *Cutibacterium* (*Propionibacterium*) *acnes*, *Streptococcus* spp., *Enterococcus* spp., and uropathogenic *E. coli*-as contributors to chronic prostatic inflammation and tumor initiation (Sfanos and De Marzo, 2012; Zhong et al., 2022; Lachance et al., 2024). The enrichment of these taxa in prostate-associated or gut microbial communities is frequently observed in the context of reduced alpha diversity and distinct beta diversity patterns, supporting the presence of compositionally altered microbial ecosystems linked to disease progression.

A clinical isolate of *E. coli*, known as CP1, has an unusual ability to persist in prostate tissue and reshape its immune environment. In experimental settings, CP1 induces a form of immunogenic cell death characterized by the exposure of calreticulin on the cell surface and the release of HMGB1 (Anker et al., 2018). These molecular signals attract cytotoxic CD8⁺ T cells, Th17 lymphocytes, dendritic cells, and M1 macrophages, while reducing regulatory T cell numbers and VEGF expression (Anker *et al*., 2018). The shift in the tumor microenvironment toward a pro-inflammatory and antigen-responsive state increases the responsiveness to PD-1 blockade (Anker *et al*., 2018; Gopalakrishnan et al., 2018; Routy et al., 2018). Together, these data suggest that specific bacterial strains can function as immune stimulants, converting otherwise immunologically inert prostate tumors into lesions capable of mounting an antitumor response.

In recent years, the link between the gut microbiota and prostate cancer has drawn increasing interest for its therapeutic implications. Experimental work suggests that antibiotics can disturb the normal gut microbiota, leading to changes that promote tumor growth and make treatments such as docetaxel, one of the key drugs used for advanced prostate cancer, less effective (Sfanos et al., 2018; Zhong et al., 2022). These antibiotic-induced perturbations are associated with reductions in microbial alpha diversity and pronounced shifts in beta diversity, reflecting loss of microbial resilience and altered community structure (Sfanos *et al*., 2018).

In contrast, interventions that help to reestablish microbial balance appear to improve both immune function and drug sensitivity. Preclinical studies using natural compounds such as icaritin and curcumol (ICA-CUR) have shown that these agents reshape the gut microbiota, leading to smaller tumor masses, decreased epithelial-mesenchymal transition, and inhibition of the DNMT1/IGFBP2 pathway involved in proliferation. Treatment with ICA-CUR also increases the presence of CD8⁺ T lymphocytes within tumors and elevates reactive oxygen species, effects consistent with stronger immune control of tumor growth, in parallel with partial restoration of microbial diversity and community composition (Xu et al., 2024).

Immunotherapy, while transformative in other solid tumors, has shown limited success in unselected prostate cancer. The microbiome might partly explain this refractoriness. Beneficial taxa such as *Akkermansia muciniphila* and *Ruminococcus* spp. have been associated with improved checkpoint inhibitor responses in other cancers and may inform patient selection or microbiome manipulation strategies to sensitize tumors to PD-1/PD-L1 blockade (Gopalakrishnan et al., 2018; Routy et al., 2018). Understanding these interactions in the context of the immunologically “cold” prostate tumor microenvironment is a promising avenue.

From a translational perspective, microbiome-informed biomarkers could refine prognostic assessment and enable more personalized treatment. Profiles enriched with *Pseudomonadota* or with increased LPS-associated signaling might predict poor chemotherapy response or aggressive disease (Sfanos et al., 2018; Zhong et al., 2022), whereas restoration of eubiotic microbial communities, characterized by higher alpha diversity and balanced beta diversity profiles, may synergize with immunotherapy and androgen receptor-targeted therapies (Lachance et al., 2024).

## Lung cancer

Lung cancer remains one of the deadliest malignancies worldwide, accounting for over 1.8 million deaths each year (Ferlay *et al*., 2024). Although tobacco smoking, environmental exposure, and inherited susceptibility are well-established risk factors, emerging research suggests that both lung and gut microbiomes may also influence how these tumors develop and progress (Karvela et al., 2023).

Advances in next-generation sequencing have changed the long-held view that healthy lungs are sterile; notably, the lungs were initially excluded from the four body sites (the gastrointestinal tract, mouth, vagina, and skin) targeted in the original goals of the Human Microbiome Project (Kiley and Caler, 2014). Using these advanced sequencing tools, small but metabolically active microbial communities have been identified in healthy lungs, mainly composed of *Bacillota*, *Bacteroidota*, and *Pseudomonadota*, including species from the genera *Prevotella*, *Streptococcus*, *Veillonella*, and *Haemophilus* (Zhao et al., 2021; Li et al., 2024a ; Emadi et al., 2025). This imbalance between the healthy lung microbiota and tumor-associated bacteria is often characterized by a decline in microbial diversity and an increase in the abundance of taxa associated with inflammation and immune dysregulation (Emadi *et al*., 2025).

Comparative analyses of lung samples consistently demonstrate that tumor-associated microbiota differ from non-malignant tissue not only in composition but also in community structure. In this context, alpha diversity and beta diversity have been systematically assessed. Several studies report reduced alpha diversity and a statistically significant separation in beta diversity analyses, indicating the presence of compositionally distinct microbial communities in cancer versus non-cancer lungs, frequently associated with depletion of health-associated genera such as *Prevotella* and *Veillonella* and relative enrichment of inflammation-associated taxa, including members of the *Proteobacteria* (Zhao et al., 2021; Emadi et al., 2025).

The oral cavity serves as a natural habitat for many microorganisms, some of which can reach the lungs through small episodes of microaspiration. Several studies have identified the periodontal pathogens, *F. nucleatum*, *Porphyromonas gingivalis*, and *Capnocytophaga* spp., in lung tumor tissues (Pathak et al., 2021). These microorganisms are often found in tumors with more aggressive features and a higher risk of metastasis. 

Oral microbiome studies further report that lower alpha diversity and altered beta diversity profiles are associated with increased lung cancer risk, particularly in prediagnostic samples, suggesting that loss of microbial richness and shifts in community composition may precede tumor development (Pathak et al., 2021). Oral microbiome studies further report that lower alpha diversity and altered beta diversity profiles are associated with increased lung cancer risk, particularly in prediagnostic samples, suggesting that loss of microbial richness and shifts in community composition may precede tumor development (Pathak *et al*., 2021). Studies indicate that previously described oral- and airway-associated bacterial communities enriched under dysbiotic conditions can activate inflammatory signaling pathways, including IL-6/STAT3 and NF-κB (Zhao et al., 2021), thereby promoting pro-inflammatory cytokine production and pattern-recognition receptor activation that sustain chronic inflammation and create a tissue environment conducive to tumor growth while impairing immune surveillance (Greathouse et al., 2018). 

Cigarette smoke alters the microbial landscape of the respiratory tract in multiple ways. It consistently reduces the abundance of beneficial commensal taxa and favors the expansion of pro-inflammatory groups, disrupting the microbiota balance that usually helps maintain airway homeostasis (Pathak et al., 2021). Smoking-associated dysbiosis has also been linked to reduced alpha diversity and to distinct beta diversity clustering in oral and respiratory samples compared with non-smokers, reflecting profound and statistically supported shifts in microbial community structure driven by tobacco exposure, often involving enrichment of genera such as *Streptococcus* and *Prevotella* in the airways (Pathak *et al*., 2021). As the epithelial lining becomes damaged, microbes adhere more readily to the mucosal surface and form biofilms, which sustain chronic inflammation and, over time, contribute to tissue injury (Li et al., 2024).

Recent studies indicate that microbial communities present in the lung tumor microenvironment can directly influence immune regulation and suppression (Nejman et al., 2020). Genera such as *Sphingomonas* and *Pseudomonas* are frequently detected in lung tumor tissue and have been linked to immunosuppressive features of the TME; notably, intratumoral fungi (e.g., *Candida* spp.) can drive myeloid-derived suppressor cells (MDSC) expansion via Dectin-1/CARD9-IL-1β signaling, favoring macrophage M2 polarization and antigen-presentation defects (Öz et al., 2016; Nejman *et al*., 2020; Liu et al., 2023). Tumor tissues also display reduced alpha diversity and a statistically significant separation in beta diversity when compared with adjacent non-tumor tissues, reflecting consistent differences in overall microbial community composition between groups rather than stochastic variation, and supporting the concept of a structurally and functionally distinct intratumoral microbial ecosystem (Nejman *et al*., 2020; Liu *et al*., 2023). As a result, regulatory T cells become more frequent while cytotoxic T cell responses weaken, creating an immune environment that allows tumor cells to persist and evade control mechanisms (Liu *et al*., 2023; Li *et al*., 2024).

Evidence has also strengthened the concept of a gut-lung axis, suggesting that intestinal microbes influence immune and metabolic processes beyond the digestive tract (Budden et al., 2017; Zhang et al., 2020). When the gut microbiota loses its balance, the intestinal barrier becomes more permeable, allowing microbial fragments and metabolites to enter the bloodstream. Once in circulation, these molecules can reach the lungs and alter local immune activity (Li et al., 2019). In lung cancer, beneficial taxa such as *Lactobacillus* and *Bifidobacterium* are often reduced, while opportunistic species, including *E. coli* and *Enterococcus*, increase in abundance (Sun et al., 2023), a pattern frequently associated with altered beta diversity despite partially preserved alpha diversity in early-stage disease (Zhang *et al*., 2020).

As microbial composition changes, signs of systemic inflammation tend to increase, and patients usually show less favorable outcomes (Jin et al., 2019; Li et al., 2024a). Several studies report that patients exhibiting lower microbial alpha diversity and more pronounced beta diversity dissimilarity show poorer clinical outcomes, linking disruption of microbial community structure to prognosis and disease severity (Jin *et al*., 2019). Circulating bacterial products, particularly LPS, have been detected in patients with lung cancer and are known to activate the NF-κB-IL6-STAT3 signaling pathway in lung tissue. This activation promotes tumor proliferation, inflammation, and resistance to chemotherapy (Hattar et al., 2013; Zhang et al., 2016; Kitamura et al., 2017; Guo et al., 2024). 

Beyond inflammation, host genetic context also influences how microbes shape lung cancer biology. Studies have shown that *TP53*-mutant tumors carry distinct microbial profiles. In squamous cell carcinoma, enrichment for *Acidovorax* spp. has been observed among smokers with *TP53* mutations, suggesting that microbial colonization might act as a promoter in lung carcinogenesis by inactivating tumor suppressor genes (Greathouse et al., 2018; Tong et al., 2024). These tumors also exhibit distinct beta diversity patterns compared with TP53-wild-type counterparts, supporting genotype-associated microbial stratification rather than random colonization (Greathouse *et al*., 2018). Multiomics analyses of large non-small cell lung carcinoma (NSCLC) cohorts further indicate that *TP53* mutations in adenocarcinomas are associated with broader alterations in microbial composition, immune infiltration, and tissue architecture than in squamous tumors (Liu et al., 2023). These findings suggest that *TP53* status and the tumor-associated microbiome may coevolve within the lung microenvironment, influencing inflammation, immune surveillance, and disease progression (Tong *et al*., 2024).

Microbial metabolites play a fundamental role in these processes. SCFAs, particularly butyrate, are generally recognized for their anti-inflammatory and epigenetic regulatory roles in the gut. However, in the context of lung cancer, their effects appear to depend on the surrounding metabolic and cellular environment. Recent analyses suggest that butyrate can modulate oncogenic signaling and non-coding RNA expression, such as *H19*, thereby influencing tumor growth and immune responses (Liu et al., 2023; Yan et al., 2025). In addition, other metabolites that accumulate during dysbiosis - including polyamines, lactate, and secondary bile acids - can promote oxidative stress, cause DNA damage, and induce epigenetic alterations that sustain malignant transformation (Greathouse et al., 2018; Guo et al., 2024). Together, these findings emphasize that microbial metabolism influences cancer not only through local interactions but also through systemic biochemical reprogramming that affects tumor behavior and progression.

Microbiome-immune interactions are particularly relevant for therapeutic outcomes. The gut microbiota strongly influences response to immune checkpoint inhibitors (ICIs). Enrichment of *Akkermansia muciniphila* and *Ruminococcus* spp. has been associated with improved PD-1 blockade efficacy in both preclinical and clinical studies, whereas broad-spectrum antibiotics reduce responsiveness (Routy et al., 2018). In patients with NSCLC, the composition of the gut microbiota appears to influence the response to immunotherapy. Those with a higher baseline abundance of beneficial bacterial groups tend to experience longer progression-free survival and fewer immune-related side effects (Jin et al., 2019). These observations have encouraged early-phase clinical trials to test whether interventions such as probiotics or fecal microbiota transplantation (FMT) could help reestablish microbial balance and improve the effectiveness of immune checkpoint inhibitors (Baruch et al., 2021).

Current evidence indicates that dysbiosis of the lung and gut microbiomes play a significant role in shaping tumor development, immune regulation, and treatment response in lung cancer. At the same time, maintaining or restoring a balanced microbial ecosystem appears to improve responses to immunotherapy and chemotherapy, suggesting that the microbiome could become a modifiable component of cancer care.

## Colorectal cancer 

Colorectal cancer (CRC) clearly demonstrates how disturbances in the gut microbiome can influence tumor initiation, progression, and response to therapy. The intestinal tract contains one of the most diverse microbial systems in the human body. These bacteria, fungi, viruses, and archaea act together to maintain the intestinal barrier, regulate immunity, and balance metabolism (Song et al., 2020). Under physiological conditions, this ecosystem maintains a symbiotic equilibrium that supports barrier integrity and anti-inflammatory signaling. When this ecosystem is disturbed by diet, obesity, antibiotics, or genetic background, microbial diversity decreases, and pro-inflammatory species begin to dominate (Louis et al., 2014; Garrett, 2019).

Dysbiosis creates a pro-inflammatory and oxidative environment that damages the intestinal barrier and promotes DNA instability. As these effects accumulate, normal epithelial cells may acquire molecular alterations that drive neoplastic transformation (Francescone et al., 2014). Among the microorganisms most consistently linked to CRC are *F. nucleatum*, enterotoxigenic *Bacteroides fragilis* (ETBF), and *E. coli* strains producing colibactin. Other bacteria, including *Enterococcus faecalis*, *Campylobacter*, *Peptostreptococcus*, *Shigella*, and *Streptococcus gallolyticus*, are also found in higher abundance in tumor samples (Thomas et al., 2019). Meanwhile, beneficial species such as *Faecalibacterium prausnitzii*, *Roseburia*, *Blautia*, and *Bifidobacterium* are often depleted, thereby reducing the production of SCFAs, such as butyrate, which protect against inflammation (Sánchez-Alcoholado et al., 2020).

*F. nucleatum*, commonly part of the oral microbiota, can also colonize the colon. This bacterium has adhesion molecules, FadA and Fap2, that interact directly with epithelial E-cadherin, setting off β-catenin signaling. This pathway promotes epithelial cell proliferation and supports tumor development (Rubinstein et al., 2013). This bacterium also interacts with Toll-like receptors (TLR2/4) and regulates microRNAs such as miR-21, increasing genomic instability and resistance to chemotherapy (Yu et al., 2017). Clinically, high levels of *F*. *nucleatum* in tumors are associated with advanced disease and a poor response to fluoropyrimidine-based therapy (Mima et al., 2016).

Another bacterium associated with CRC, the ETBF, secretes a zinc-dependent metalloprotease, fragilysin (BFT), which cleaves E-cadherin and disrupts epithelial integrity. As a result, key oncogenic pathways, including WNT/β-catenin, NF-κB, STAT3, and NOTCH, are activated, promoting sustained inflammation and cell proliferation (Wu et al., 2009). Chronic exposure to ETBF has been shown to drive IL17-mediated inflammation and impair apoptosis, thereby creating conditions that benefit tumor initiation and progression (Boleij et al., 2015).

Certain *E. coli* strains contribute to colorectal carcinogenesis through direct genotoxicity. The pks genomic island (*polyketide synthase genomic island*) encodes colibactin. This polyketide-nonribosomal peptide hybrid induces DNA double-strand breaks, leaving a mutational signature detectable in human CRC genomes (Pleguezuelos-Manzano et al., 2020). The pk genomic island is a gene group present in some *E. coli* strains, especially *E. coli* type B group, which are commensal opportunistic bacteria that can become pathogens under certain conditions (Desvaux et al., 2020). This evidence provides one of the most direct mechanistic links between microbial activity and somatic mutation in human cancer. At the same time, *Enterococcus faecalis* can generate reactive oxygen species that inflict oxidative damage on host DNA, contributing to mutagenesis and genomic instability. Other bacterias, including *Campylobacter* spp. and *Streptococcus gallolyticus*, promote tumor growth by intensifying cytokine production and stimulating angiogenesis within the colonic mucosa (Huycke et al., 2002; Kostic et al., 2012).

Beyond bacteria, fungal and viral communities, the mycobiome and virome modulate colorectal carcinogenesis through complex interkingdom interactions. Fungi such as *Candida albicans* and *Malassezia* spp. are enriched in tumor tissues and activate pattern recognition receptors (PRRs), including Dectin-1 and TLRs, stimulating pro-inflammatory cytokine production (Coker et al., 2019). Bacteriophages also influence bacterial population dynamics and horizontal gene transfer, thereby modulating the functional potential of the gut ecosystem (Hannigan et al., 2018).

Metagenomic studies indicate that, in CRC, microbial diversity exhibits distinct behaviors across the bacterial and fungal compartments, with beta diversity emerging as a more consistent marker of dysbiosis than alpha diversity (Costa et al., 2022). In a cohort of 165 individuals (73 CRC patients and 92 controls), Coker et al. (2019) demonstrated that the bacterial microbiota shows reduced alpha diversity, whereas beta diversity clearly distinguishes patients from controls; in the intestinal mycobiome, although fungal alpha diversity does not differ between groups, beta diversity robustly discriminates CRC from controls and reveals stage-dependent stratification. Complementarily, Thomas et al. (2019), in an integrated metagenomic analysis of 969 fecal metagenomes from multiple independent cohorts, observed that bacterial alpha diversity varies across studies, whereas beta diversity consistently differentiates CRC patients from healthy individuals. Taken together, these findings indicate that CRC-associated dysbiosis is more closely related to the structural and functional reorganization of the intestinal microbiota, characterized by enrichment of potentially pro-tumorigenic microorganisms, such as *F. nucleatum*, ETBF and *E. coli*, in addition to the concomitant depletion of beneficial commensals, rather than by uniform alterations in overall microbial diversity.

Beyond taxonomic shifts, the functional consequences of dysbiosis are largely mediated by microbial metabolites. Among them, SCFAs, such as butyrate, are central to intestinal homeostasis. Butyrate is produced by fiber-fermenting bacteria such as *Faecalibacterium prausnitzii* and *Roseburia*, and it acts as a histone deacetylase (HDAC) inhibitor. This epigenetic regulation keeps chromatin in an open state, promoting the expression of genes involved in cell differentiation, apoptosis, and DNA repair, while limiting uncontrolled proliferation (Louis et al., 2014).

Butyrate also serves as a primary energy source for colonocytes and reinforces the intestinal barrier by stabilizing tight junctions and promoting mucin production. At the same time, it dampens NF-κB signaling and supports the expansion of regulatory T cells, sustaining an anti-inflammatory environment (Recharla et al., 2023). In colorectal cancer, however, this balance is disturbed. The decline in butyrate-producing bacteria leads to weakened HDAC inhibition and barrier function, creating conditions that favor persistent inflammation and genomic instability (Fang et al., 2021). In advanced colorectal cancer, metabolic reprogramming in tumor cells may also limit the ability to utilize butyrate as an energy source, altering its physiological role. Under these conditions, butyrate may even reinforce tumor growth rather than suppress it, highlighting the complex, context-dependent nature of microbial metabolism in cancer (Han et al., 2018). 

Meanwhile, secondary bile acids and polyamines, often increased in dysbiosis, stimulate epithelial proliferation, DNA replication, and WNT pathway activation (Ridlon et al., 2014). Persistent exposure to microbial LPS and peptidoglycan sustains NF-κB and STAT3 signaling, establishing a feed-forward loop between inflammation and tumor growth (Greathouse et al., 2018).

The immunological effects of dysbiosis are not confined to the intestinal mucosa. Microorganisms that thrive within tumors can shape the local immune landscape by attracting MDSCs and tumor-associated macrophages (TAMs), dampening antigen presentation, and suppressing cytotoxic T cell activity. These changes collectively create conditions that favor immune escape and sustain tumor growth (Francescone et al., 2014; Yu et al., 2017). In contrast, restoring a balanced microbial environment has been associated with improved immune responses. Evidence from clinical and preclinical studies suggests that interventions such as fecal microbiota transplantation, probiotic supplementation, and dietary enrichment with fermentable fiber may help restore eubiosis and enhance the efficacy of immunotherapy (Routy et al., 2023). Patients with higher baseline abundance of *Faecalibacterium* and *Ruminococcus* exhibit better responses to PD-1 blockade, whereas enrichment of *F. nucleatum* predicts resistance (Gopalakrishnan et al., 2018; Routy *et al*., 2018).

Microbiota composition also affects conventional treatments. Antibiotic exposure before or during immune checkpoint therapy reduces patient survival (Routy et al., 2018), and *F. nucleatum* predicts poor chemotherapy response (Mima et al., 2016). Evidence from clinical and preclinical studies suggests that interventions such as probiotic supplementation, dietary enrichment with fermentable fiber, and other microbiome‐modulating strategies may help restore microbial balance and enhance the efficacy of immunotherapy (Pei et al., 2024).

Although research on the colorectal microbiome has advanced considerably, many aspects remain unclear. Most current studies are descriptive and cross-sectional, making it difficult to determine whether dysbiosis acts as a driver or a byproduct of tumorigenesis. Some emerging evidence suggests that microbial metabolites, immune modulation, and epithelial interactions work together to influence cancer initiation and therapeutic response (Francescone et al., 2014; Garrett, 2019; Pleguezuelos-Manzano et al., 2020).

## Cervical cancer

Cervical cancer (CC) is probably one of the best examples of how alterations of the microbiota may lead to carcinogenesis. Although not always considered or analyzed, viruses are part of this community and, in the case of CC, the Human Papilloma Virus (HPV) is recognized as its causal agent (IARC, 2007). Currently, HPV subtypes are classified in high- or low-risk groups, according to their potential to cause CC (IARC, 2007). However, the scenery is not as simple as it may seem. HPV infections are common throughout life, even with high-risk subtypes (de Villiers et al., 1987). In this context, the persistence of the infection is reported as the key factor contributing to the development of cervical dysplasia and to its progression to CC. However, the causes of HPV persistence remain unclear (Moscicki et al., 1998). Therefore, other factors or agents are likely to play a role in this transformation process, such as other components of the microbiota.

While high bacterial diversity is a sign of health in other mucosa (Kyrgiou and Moscicki, 2022), in the cervicovaginal mucosa, it may indicate imbalance and disease. A recent meta-analysis reported that 9 of 15 studies showed higher alpha diversity, the microbial diversity within a single sample, in HPV-positive relative to HPV-negative women. Most of these studies were conducted in Asian populations and also showed a higher alpha diversity among high-risk HPV (hrHPV)-positive women relative to healthy women. Alpha diversity was also higher in CC patients relative to HPV-positive women and to healthy women (Zhang et al., 2025). A different meta-analysis showed similar results, with higher alpha diversity in CC patients relative to controls (Zhang *et al*., 2025). In a meta-analysis comprising six studies with *16S rRNA* sequencing data for 507 samples, microbial diversity was shown to be similar between cervical intraepithelial neoplasia (CIN) and CC, but higher than in normal controls and HPV-positive samples Regarding beta diversity, which reflects how distinct microbial profiles are across different samples or conditions, the results were less clear for HPV infection, with 8 of 17 studies showing differences between HPV-positive and HPV-negative/healthy women. However, in CC, two meta-analyses reported that most studies (13/16 and 19/25, respectively) showed a significant difference in beta diversity between patients and controls (Zhang *et al*., 2025). The structure of microbial communities was also shown to differ significantly between normal controls, HPV-positive, CIN, and CC samples (Li et al., 2024a).

The healthy cervicovaginal microbiota is largely composed of *Lactobacillus* spp., which seem to protect the mucosa against infections by promoting its acidification, by creating biofilms, and by producing antimicrobial compounds (Ravel et al., 2011; Anahtar et al., 2015). In this context, different studies have shown that decreased representation of *Lactobacillus* spp. in cervical, cervicovaginal, and vaginal samples is consistent in CC patients (Wen et al., 2025). Additionally, the presence of *Lactobacillus* spp. in the cervicovaginal mucosa was associated with reduced detection of hrHPV and CC in a meta-analysis including 11 studies. These associations seem to involve the presence of *L. crispatus*, but *not L. iners*, for which significant results were not found (Wang et al., 2019). 

The vaginal microbiota has also been characterized based on community state types (CST), being mainly *Lactobacillus*-enriched CST (including those dominated by *L. crispatus*, *L. gasseri*, *L. iners*, *or L. jensenii*) and *Lactobacillus*-depleted CST (with low contribution of *Lactobacillus* spp. and increased diversity of strictly anaerobic bacteria) (Ravel et al., 2011). Low-*Lactobacillus* vaginal microbiota was significantly associated with HPV infection in a meta-analysis compiling 20 studies, with an overall effect size of 1.53 (95% CI 1.23-1.82) (Tamarelle et al., 2019). Corroborating this, another meta-analysis showed a high association of *Lactobacillus*-depleted CST with the infection by any HPV when compared with *L. crispatus*-dominant CST. The association was also significant for *L. iners*-dominant CST relative to *L. crispatus*-dominant CST. When considering hrHPV, the groups with the highest risk for infection were *L. gasseri*-dominant CST, *Lactobacillus*-depleted CST, and *L. iners*-dominant CST, always relative to *L. crispatus*-dominant CST. In the same study, the association with dysplasia and cancer was also assessed, showing that *Lactobacillus*-depleted CST and *L. iners*-dominant CST present a higher risk relative to *L. crispatus*-dominant CST (Norenhag et al., 2020). With the reduction of *Lactobacillus* spp., during CC development and progression, other genera become more abundant. Among them, we may highlight *Gardnerella*, *Prevotella*, and *Atopobium*, which are not only found in bacterial vaginosis but are also reported to be more common in HPV-positive and CIN samples (Li et al., 2024) and to be associated with HPV persistence (Brotman et al., 2014; di Paola *et al*., 2017). At the same time, *L. crispatus* abundance has been associated with HPV negativity and clearance and *L. gasseri* with fastest clearance (Brotman *et al*., 2014; di Paola *et al*., 2017).

In general, studies evaluating alterations in the microbiota composition during cervical carcinogenesis steps point to a common dynamic. Alpha diversity seems to increase, especially from HPV-positive to CIN, beta diversity differs among the steps, *Actinobacillus* spp. abundance decreases, and other genera take over the cervicovaginal microenvironment. Based on this, an attempt was made to build models based on the microbiota composition to differentiate each step (Li et al., 2024 b ). Although with a relatively low accuracy (0.6568; 95% CI 0.60-0.71), *Prevotella*, *Acinetobacter*, and *Shuttleworthella* abundance were able to differentiate HPV-positive from healthy controls. The accuracy of the model to differentiate CIN from healthy controls was higher (0.7673; 95% CI 0.70-0.83) and included *Lactobacillus*, *Pseudomonas*, and *Acinetobacter*. Finally, CC was differentiated from healthy controls based on the abundance of Streptococcus, *Fusobacterium*, *Pseudomonas*, *Anaerococcus* and *Acinetobacter* with an accuracy of 0.8947 (95% CI 0.83-0.95).

At this point, it is clear that the cervicovaginal microbiota composition varies during cervical cancer development and progression. However, it is less clear whether these variations are the cause or a consequence of the carcinogenic process. Although still speculative, the knowledge of healthy microbiota, together with the findings reported here, provides a hint at the most biologically plausible mechanisms. With a reduction in the abundance of *Lactobacillus* spp., the production of protective biofilms is likely to decrease as well, making the epithelium more vulnerable to damage (Delgado-Diaz et al., 2022; Liu et al., 2024). Since HPV infects cells of the basal layer of the epithelium, such vulnerability may provide access (Audirac-Chalifour et al., 2016). At the same time, the acidification promoted by *Lactobacillus* spp., as well as the production of antimicrobial compounds, is also reduced, favoring the colonization of the microenvironment by other genera (Gajer et al., 2012; di Paola *et al*., 2017; Lebeau et al., 2022). The higher diversity, as well as the higher abundance of specific genera, such as *Atopobium*, may contribute to persistence, a key factor to transformation (di Paola *et al*., 2017). However, only longitudinal, mechanistic studies will prove whether this hypothesis holds. Additionally, if this mechanism contributes to CC development and progression, a gap remains in understanding what causes alterations in the microbiota in the first place.

## Final considerations and perspectives

Across breast, prostate, lung, colorectal, and cervical cancers, a consistent picture is emerging: the microbiome is not a peripheral bystander but a biologically active component of tumor ecosystems. Perturbations in microbial composition and function, or dysbiosis, can amplify chronic inflammation, rewire cell signaling, and modulate antitumor immunity, thereby shaping carcinogenesis, disease course, and response to therapy (Garrett, 2019; Helmink et al., 2019). Molecular mediators include microbiota-derived metabolites (e.g., short-chain fatty acids, secondary bile acids), pathogen-associated molecules such as lipopolysaccharide, and *bona fide* genotoxins, such as colibactin, which leaves a characteristic mutational footprint in human colorectal tumors (Ridlon et al., 2014; Pleguezuelos-Manzano et al., 2020).

**Figure 3 -:**
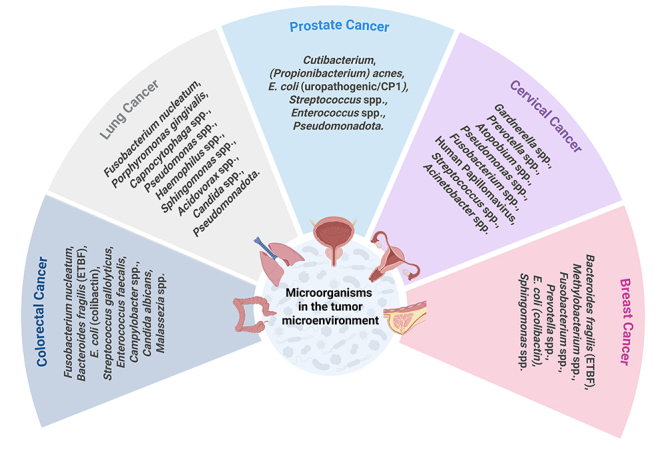
Microorganisms associated with major human cancers. Representative microbial taxa identified in tumor or adjacent tissues from five cancer types: breast, cervical, prostate, lung, and colorectal. The listed microorganisms include bacteria, fungi, and viruses that have been reported to influence carcinogenesis through inflammation, genotoxicity, metabolic modulation, and immune regulation (created in BioRender, https://BioRender.com/bqxjy55).

On the immune axis, commensal taxa such as *Akkermansia* and *Ruminococcus* have been associated with improved outcomes under immune checkpoint blockade, whereas antibiotic-driven depletion of the gut microbiota has been associated with weaker responses and inferior survival (Gopalakrishnan et al., 2018; Routy et al., 2018). Currently, no single microbial taxon can be considered a definitive biomarker for prognosis or treatment response; rather, the most promising signals arise from context-dependent, composite microbial signatures integrating taxonomic composition, community structure (including beta diversity), and functional features. Tumor-proximal readouts, such as intratumoral microbiome profiles and circulating microbial DNA, have shown potential for prognostic stratification and recurrence risk prediction, while enrichment of inflammation-associated taxa has been linked to adverse outcomes in specific clinical contexts (Greathouse et al., 2018; Nejman et al., 2020; Pathak et al., 2021; Liu et al., 2023; Sun et al., 2023). Taken together, these data argue that microbial ecology is a modifiable dimension of cancer biology with diagnostic and therapeutic value (Figure 3; Table 1).

**Table 1-:** Microorganisms influencing carcinogenesis and tumor immunity in five major cancers.

Cancer Type	Functional Group	Main Microorganisms	Mechanism / Effect	References
Breast	Protective/Anti-inflammatory	*Sphingomonas* spp., *Bifidobacterium* spp., *Ligilactobacillus* spp., *Akkermansia muciniphila*, SCFA-producing taxa (*Faecalibacterium, Roseburia*)	Maintain epithelial integrity, regulate inflammation, and enhance therapy response.	Xuan *et al*., 2014; Banerjee et al., 2015; Gopalakrishnan et al., 2018; Routy et al., 2018; Peng et al., 2024.
	Pro-tumoral/Pro-inflammatory	*Methylobacterium* spp., *Fusobacterium* spp., *Prevotella* spp., *E. coli* (colibactin), *Bacteroides fragilis* (ETBF), LPS- and β-glucuronidase-producing taxa	Promote inflammation, DNA damage, estrogen reactivation, and oncogenic signaling (Notch, β-catenin, TLR4).	Xuan *et al*., 2014; Banerjee et al., 2015; Nougayrède et al., 2006; Kwa et al., 2016; Parida et al., 2021.
Prostate	Protective/Anti-inflammatory	*Lactobacillus* spp., *Bifidobacterium* spp., *Akkermansia muciniphila*, *Ruminococcus* spp.	Support immune regulation, maintain gut-prostate balance, enhance immunotherapy.	Lachance et al., 2024; Gopalakrishnan et al., 2018; Routy et al., 2018.
	Pro-tumoral/Pro-inflammatory	*Cutibacterium* (*Propionibacterium*) *acnes*, *Streptococcus* spp., *Enterococcus* spp., *E. coli* (uropathogenic/CP1), *Pseudomonadota*	Induce chronic inflammation, DNA damage, NF-κB-IL6-STAT3 activation, and chemoresistance.	Sfanos and De Marzo, 2012; Zhong et al., 2022; Lachance et al., 2024; Anker et al., 2018.
Lung	Protective/Anti-inflammatory	*Lactobacillus* spp., *Bifidobacterium* spp., *Akkermansia muciniphila*, *Ruminococcus* spp.	Regulate immune tone; improve checkpoint inhibitor response.	Jin et al., 2019; Matson *et al*., 2018; Routy et al., 2018.
	Pro-tumoral/Pro-inflammatory	*Fusobacterium nucleatum*, *Porphyromonas gingivalis*, *Capnocytophaga* spp., *Pseudomonas* spp., *Haemophilus* spp., *Sphingomonas* spp., *Acidovorax* spp., *Candida* spp., *Pseudomonadota*	Trigger IL6/STAT3 and NF-κB signaling; promote MDSC expansion and immunosuppression; linked to *TP53* mutation.	Greathouse et al., 2018; Nejman et al., 2020; Liu et al., 2023; Tong et al., 2024; Li *et al*., 2024; Emadi et al., 2025.
Colorectal	Protective/Anti-inflammatory	*Faecalibacterium prausnitzii*, *Roseburia* spp., *Blautia* spp., *Bifidobacterium* spp., *Ruminococcus* spp.	Produce butyrate; sustain barrier integrity and anti-inflammatory signaling.	Sánchez-Alcoholado et al., 2020; Recharla et al., 2023; Routy et al., 2018.
	Pro-tumoral/Pro-inflammatory	*Fusobacterium nucleatum*, *Bacteroides fragilis* (ETBF), *E. coli* (colibactin), *Enterococcus faecalis*, *Campylobacter* spp., *Streptococcus gallolyticus*, *Candida albicans*, *Malassezia* spp.	Promote inflammation, DNA damage, β-catenin/NF-κB/STAT3 activation, immune evasion, and drug resistance.	Wu et al., 2009; Rubinstein et al., 2013; Yu et al., 2017; Coker et al., 2019; Pleguezuelos-Manzano et al., 2020; Spencer *et al*., 2021.
Cervical	Protective/Anti-inflammatory	*Lactobacillus* spp. (*L. crispatus*, *L. gasseri*, *L. jensenii*)	Maintain mucosal acidity and integrity; produce antimicrobial compounds; associated with HPV clearance.	Ravel et al., 2011; Anahtar et al., 2015; Brotman et al., 2014; di Paola *et al*., 2017; Norenhag et al., 2020.
	Pro-tumoral/Pro-inflammatory	*Gardnerella* spp., *Prevotella* spp., *Atopobium* spp., *Pseudomonas* spp., *Fusobacterium* spp., *Acinetobacter* spp., *Streptococcus* spp., *Human Papillomavirus*	Promote dysbiosis, inflammation, and HPV persistence; linked to CIN and cancer progression.	Li et al., 2024; Zhang et al., 2025; Brotman et al., 2014; di Paola *et al*., 2017.

Translating this knowledge into practice will require parallel advances in measurement, mechanisms, and interventions. Measurement: low-biomass tissues (e.g., breast and lung) are vulnerable to reagent and environmental contamination, and study-to-study variability in sampling, extraction, and bioinformatics pipelines remains a major barrier to reproducibility. Stringent negative controls, quantitative validation, and decontamination routines are essential; equally important is protocol harmonization across cohorts (Eisenhofer et al., 2019). 

The characterization of the tumor microbiome relies mainly on three complementary approaches: *16S rRNA* gene sequencing, shotgun metagenomics, and, more recently, spatial profiling. *16S rRNA* sequencing is widely used due to its sensitivity and lower cost, being particularly suitable for low-biomass samples; however, it offers limited taxonomic resolution and is highly susceptible to contamination, requiring rigorous negative controls and computational decontamination strategies (Eisenhofer et al., 2019). Shotgun metagenomics offers higher taxonomic and functional resolution, enabling the identification of microbial genes and their associated metabolic pathways. In addition to providing a more comprehensive characterization of the microbiome, this approach allows the simultaneous profiling of multiple taxonomic groups, including bacteria, archaea, viruses, and fungi, which are increasingly recognized as functionally relevant components of the tumor-associated microbiome. However, this methodology requires higher DNA input and stringent quality control, particularly when applied to low-biomass tumor tissues (Nejman et al., 2020). Spatial profiling preserves tissue architecture and allows discrimination between tissue-associated microorganisms and environmental contaminants, although its application is still limited by cost and technical complexity (Nejman *et al*., 2020; Liu et al., 2023). Integrating these approaches, together with standardized protocols and appropriate controls, represents the best current practice in tumor microbiome research.

An important source of variability across microbiome-cancer studies lies in differences in study design, sample size, geographic origin, and methodological approaches. Many investigations rely on relatively small and heterogeneous cohorts, often drawn from distinct populations with different dietary patterns, environmental exposures, ancestry, and healthcare access, all of which are known to influence baseline microbiota composition (Rothschild et al., 2018; Garrett, 2019). In addition, variability in sample type (stool, tissue, blood, or mucosal swabs), sequencing strategies (*16S rRNA* gene profiling versus shotgun metagenomics), DNA extraction protocols, and bioinformatic pipelines contributes substantially to inconsistencies across studies, particularly in low-biomass tissues such as breast and lung (Eisenhofer et al., 2019; Nejman et al., 2020). These factors limit direct comparability and may partially explain discordant findings reported in the literature.

Another major challenge is distinguishing causality from association. Most available human evidence remains observational and descriptive, making it difficult to determine whether observed microbial alterations actively drive carcinogenesis or instead reflect secondary changes induced by the tumor microenvironment, inflammation, or therapy. Although mechanistic insights have emerged from experimental models, such as genotoxin-induced mutational signatures in colorectal cancer or microbiome-mediated modulation of immune responses, establishing cause-effect relationships in humans remains a critical gap (Garrett, 2019; Pleguezuelos-Manzano et al., 2020). Longitudinal, multi-omics studies and functional validation in controlled systems will be essential to move beyond correlative associations.

In addition to methodological heterogeneity, biological differences among cancer types and histological subtypes further complicate microbiota-cancer associations. Tumors arising from distinct epithelial lineages, such as adenocarcinomas, squamous cell carcinomas, small cell carcinomas, sarcomas, or transitional cell carcinomas, differ markedly in tissue architecture, metabolic demands, immune infiltration, and stromal composition. These intrinsic differences likely shape microbial colonization, persistence, and functional impact within the tumor microenvironment. For example, prostate cancer encompasses multiple histological and molecular entities, each associated with distinct inflammatory and immune landscapes, which may influence how microbial signals are integrated at the tissue level (Sfanos and De Marzo, 2012; Lachance et al., 2024). Failure to account for such heterogeneity may obscure subtype-specific microbiome signatures and limit the translational relevance of microbiome-based biomarkers.

Clinical implementation is likely to proceed along two complementary tracks. One is biomarker development, where stool, saliva, or minimally invasive tissue assays could augment genetic and epigenetic risk models for early detection, prognosis, and therapy selection. Here, companion diagnostics that integrate microbial signatures with tumor genomics and circulating metabolites may offer the greatest leverage (Garrett, 2019). The second emerging direction involves microbiome-guided therapies, which range from supportive strategies, such as high-fiber nutrition and responsible antibiotic use, to next-generation live biotherapeutics designed to release immunoregulatory or metabolic compounds directly within the tumor environment (Helmink et al., 2019). Among these approaches, fecal microbiota transplantation has already demonstrated proof of concept in early-phase clinical trials, particularly in melanoma patients refractory to immune checkpoint inhibitors, supporting the translational relevance of microbiome modulation in oncology (Routy et al., 2018; Baruch et al., 2021). As these strategies advance, keeping equity remains critical. Ancestry, geographic context, diet, and prior antibiotic exposure all influence the homeostasis of the resident microbiota and may affect both the accuracy of microbial biomarkers and the effectiveness of microbiome-based treatments. This highlights the importance of studies in diverse and well-characterized populations (Rothschild et al., 2018).

In short, the microbiome offers a tractable layer of biology that can be measured, modeled, and, crucially, modified. Realizing its clinical promise will require rigor in low-biomass sampling, standardized analytics, integrative multi-omics, and carefully designed intervention trials. As these scientific domains converge, microbial ecology will cease to be only descriptive and may stand as a proper foundation for precision oncology. However, progress in this field must unfold with fairness and responsibility. The human microbiota reflects ancestry, environment, diet, and past antibiotic exposure, factors that can influence both the reliability of microbial biomarkers and how patients respond to microbiome-based therapies.

## Data Availability

This article is a literature review and does not include new data.

## References

[B1] Abdul Nazeer H, Kannan S, Balakrishanan J, Nair VK, Kavitha Y, Bhosale NK (2025). The gut microbiota and breast cancer: A comprehensive review of
emerging links and therapeutic implications. Med Microecol.

[B2] Anahtar MN, Byrne EH, Doherty KE, Bowman BA, Yamamoto HS, Soumillon M, Padavattan N, Ismail N, Moodley A, Sabatini ME (2015). Cervicovaginal bacteria are a major modulator of host
inflammatory responses in the female genital tract. Immunity.

[B3] Anker JF, Naseem AF, Mok H, Schaeffer AJ, Abdulkadir SA, Thumbikat P (2018). Multi-faceted immunomodulatory and tissue-tropic clinical
bacterial isolate potentiates prostate cancer immunotherapy. Nat Commun.

[B4] Audirac-Chalifour A, Torres-Poveda K, Bahena-Román M, Téllez-Sosa J, Martínez-Barnetche J, Cortina-Ceballos B, López-Estrada G, Delgado-Romero K, Burguete-García AI, Cantú D (2016). Cervical microbiome and cytokine profile at various stages of
cervical cancer: A pilot study. PLoS One.

[B5] Banerjee S, Wei Z, Tan F, Peck KN, Shih N, Feldman M, Rebbeck TR, Alwine JC, Robertson ES (2015). Distinct microbiological signatures associated with triple
negative breast cancer. Sci Rep.

[B6] Baruch EN, Youngster I, Ben-Betzalel G, Ortenberg R, Lahat A, Katz L, Adler K, Dick-Necula D, Raskin S, Bloch N (2021). Fecal microbiota transplant promotes response in
immunotherapy-refractory melanoma patients. Science.

[B7] Berg G, Rybakova D, Fischer D, Cernava T, Vergès MC, Charles T, Chen X, Cocolin L, Eversole K, Corral GH (2020). Microbiome definition re-visited: Old concepts and new
challenges. Microbiome.

[B8] Boleij A, Hechenbleikner EM, Goodwin AC, Badani R, Stein EM, Lazarev MG, Ellis B, Carroll KC, Albesiano E, Wick EC (2015). The Bacteroides fragilis toxin gene is prevalent in the colon
mucosa of colorectal cancer patients. Clin Infect Dis.

[B9] Boshart M, Gissmann L, Ikenberg H, Kleinheinz A, Scheurlen W, zur Hausen H (1984). A new type of papillomavirus DNA, its presence in genital cancer
biopsies and in cell lines derived from cervical cancer. EMBO J.

[B10] Brotman RM, Shardell MD, Gajer P, Tracy JK, Zenilman JM, Ravel J, Gravitt PE (2014). Interplay between the temporal dynamics of the vaginal microbiota
and human papillomavirus detection. J Infect Dis.

[B11] Budden KF, Gellatly SL, Wood DL, Cooper MA, Morrison M, Hugenholtz P, Hansbro PM (2017). Emerging pathogenic links between microbiota and the gut-lung
axis. Nat Rev Microbiol.

[B12] Castellarin M, Warren RL, Freeman JD, Dreolini L, Krzywinski M, Strauss J, Barnes R, Watson P, Allen-Vercoe E, Moore RA, Holt RA (2012). Fusobacterium nucleatum infection is prevalent in human
colorectal carcinoma. Genome Res.

[B13] Cavarretta I, Ferrarese R, Cazzaniga W, Saita D, Lucianò R, Ceresola ER, Locatelli I, Visconti L, Lavorgna G, Briganti A (2017). The microbiome of the prostate tumor
microenvironment. Eur Urol.

[B14] Coker OO, Nakatsu G, Dai RZ, Wu WKK, Wong SH, Ng SC, Chan FKL, Sung JJY, Yu J (2019). Enteric fungal microbiota dysbiosis and ecological alterations in
colorectal cancer. Gut.

[B15] Costa CPD, Vieira P, Mendes-Rocha M, Pereira-Marques J, Ferreira RM, Figueiredo C (2022). The tissue-associated microbiota in colorectal cancer: A
systematic review. Cancers (Basel).

[B16] Cullin N, Azevedo Antunes C, Straussman R, Stein-Thoeringer CK, Elinav E (2021). Microbiome and cancer. Cancer Cell.

[B17] de Villiers EM, Wagner D, Schneider A, Wesch H, Miklaw H, Wahrendorf J, Papendick U, zur Hausen H (1987). Human papillomavirus infections in women with and without
abnormal cervical cytology. Lancet.

[B18] Delgado-Diaz DJ, Jesaveluk B, Hayward JA, Tyssen D, Alisoltani A, Potgieter M, Bell L, Ross E, Iranzadeh A, Allali I (2022). Lactic acid from vaginal microbiota enhances cervicovaginal
epithelial barrier integrity by promoting tight junction protein
expression. Microbiome.

[B19] Dent R, Trudeau M, Pritchard KI, Hanna WM, Kahn HK, Sawka CA, Lickley LA, Rawlinson E, Sun P, Narod SA (2007). Triple-negative breast cancer: clinical features and patterns of
recurrence. Clin Cancer Res.

[B20] Desvaux M, Dalmasso G, Beyrouthy R, Barnich N, Delmas J, Bonnet R (2020). Pathogenicity factors of genomic islands in intestinal and
extraintestinal Escherichia coli. Front Microbiol.

[B21] Di Paola M, Sani C, Clemente AM, Iossa A, Perissi E, Castronovo G, Tanturli M, Rivero D, Cozzolino F, Cavalieri D (2017). Characterization of cervico-vaginal microbiota in women
developing persistent high-risk human papillomavirus
infection. Sci Rep.

[B22] Dürst M, Gissmann L, Ikenberg H, zur Hausen H (1983). A papillomavirus DNA from a cervical carcinoma and its prevalence
in cancer biopsy samples from different geographic regions. Proc Natl Acad Sci U S A.

[B23] Eisenhofer R, Minich JJ, Marotz C, Cooper A, Knight R, Weyrich LS (2019). Contamination in low microbial biomass microbiome studies: Issues
and recommendations. Trends Microbiol.

[B24] Emadi R, Saki S, Yousefi P, Tabibzadeh A (2025). A perspective on lung cancer and lung microbiome: Insight on
immunity. Immun Inflamm Dis.

[B25] Epstein MA, Achong BG, Barr YM (1964). Virus particles in cultured lymphoblasts from Burkitt’s
lymphoma. Lancet.

[B26] Fang Y, Yan C, Zhao Q, Xu J, Liu Z, Gao J, Zhu H, Dai Z, Wang D, Tang D (2021). The roles of microbial products in the development of colorectal
cancer: A review. Bioengineered.

[B27] Francescone R, Hou V, Grivennikov SI (2014). Microbiome, inflammation, and cancer. Cancer J.

[B28] Garrett WS (2019). The gut microbiota and colon cancer. Science.

[B29] Gajer P, Brotman RM, Bai G, Sakamoto J, Schütte UM, Zhong X, Koenig SS, Fu L, Ma ZS, Zhou X (2012). Temporal dynamics of the human vaginal microbiota. Sci Transl Med.

[B30] Gopalakrishnan V, Spencer CN, Nezi L, Reuben A, Andrews MC, Karpinets TV, Prieto PA, Vicente D, Hoffman K, Wei SC (2018). Gut microbiome modulates response to anti-PD-1 immunotherapy in
melanoma patients. Science.

[B31] Greathouse KL, White JR, Vargas AJ, Bliskovsky VV, Beck JA, von Muhlinen N, Polley EC, Bowman ED, Khan MA, Robles AI (2018). Interactions between the microbiome and TP53 in human lung
cancer. Genome Biol.

[B32] Guo Q, Jin Y, Chen X, Ye X, Shen X, Lin M, Zeng C, Zhou T, Zhang J (2024). NF-κB in biology and targeted therapy: New insights and
translational implications. Signal Transduct Target Ther.

[B33] Han A, Bennett N, Ahmed B, Whelan J, Donohoe DR (2018). Butyrate decreases its own oxidation in colorectal cancer cells
through inhibition of histone deacetylases. Oncotarget.

[B34] Hanahan D (2022). Hallmarks of cancer: New dimensions. Cancer Discov.

[B35] Hannigan GD, Duhaime MB, 4th Ruffin MT, Koumpouras CC, Schloss PD (2018). Diagnostic potential and interactive dynamics of the colorectal
cancer virome. MBio.

[B36] Hattar K, Savai R, Subtil FS, Wilhelm J, Schmall A, Lang DS, Goldmann T, Eul B, Dahlem G, Fink L (2013). Endotoxin induces proliferation of NSCLC in vitro and in vivo:
Role of COX-2 and EGFR activation. Cancer Immunol Immunother.

[B37] Helmink BA, Khan MAW, Hermann A, Gopalakrishnan V, Wargo JA (2019). The microbiome, cancer, and cancer therapy. Nat Med.

[B38] Hieken TJ, Chen J, Hoskin TL, Walther-Antonio M, Johnson S, Ramaker S, Xiao J, Radisky DC, Knutson KL, Kalari KR (2016). The microbiome of aseptically collected human breast tissue in
benign and malignant disease. Sci Rep.

[B39] Human Microbiome Project Consortium (2012). Structure, function, and diversity of the healthy human
microbiome. Nature.

[B40] Huycke MM, Abrams and Moore DR (2002). Enterococcus faecalis produces extracellular superoxide and
hydrogen peroxide that damages colonic epithelial cell DNA. Carcinogenesis.

[B41] IARC - International Agency for Research on Cancer, Working Group on the Evaluation of Carcinogenic Risks to
Humans (2007). Human papillomaviruses. IARC Monogr Eval Carcinog Risks Hum.

[B42] Jin C, Lagoudas GK, Zhao C, Bullman S, Bhutkar A, Hu B, Ameh S, Sandel D, Liang XS, Mazzilli S (2019). Commensal microbiota promote lung cancer development via γδ T
Cells. Cell.

[B43] Karvela A, Veloudiou OZ, Karachaliou A, Kloukina T, Gomatou G, Kotteas E (2023). Lung microbiome: An emerging player in lung cancer pathogenesis
and progression. Clin Transl Oncol.

[B44] Kiley J, Caler E (2014). The lung microbiome: A new frontier in pulmonary
medicine. Ann Am Thorac Soc.

[B45] Kitamura H, Ohno Y, Toyoshima Y, Ohtake J, Homma S, Kawamura H, Takahashi N, Taketomi A (2017). Interleukin-6/STAT3 signaling as a promising target to improve
the efficacy of cancer immunotherapy. Cancer Sci.

[B46] Kostic AD, Gevers D, Pedamallu CS, Michaud M, Duke F, Earl AM, Ojesina AI, Jung J, Bass AJ, Tabernero J (2012). Genomic analysis identifies association of Fusobacterium with
colorectal carcinoma. Genome Res.

[B47] Kustrimovic N, Bombelli R, Baci D, Mortara L (2023). Microbiome and prostate cancer: A novel target for prevention and
treatment. Int J Mol Sci.

[B48] Kwa M, Plottel CS, Blaser MJ, Adams S (2016). The intestinal microbiome and estrogen receptor-positive female
breast cancer. J Natl Cancer Inst.

[B49] Kyrgiou M, Moscicki AB (2022). Vaginal microbiome and cervical cancer. Semin Cancer Biol.

[B50] Lachance G, Robitaille K, Laaraj J, Gevariya N, Varin TV, Feldiorean A, Gaignier F, Julien IB, Xu HW, Hallal T (2024). The gut microbiome-prostate cancer crosstalk is modulated by
dietary polyunsaturated long-chain fatty acids. Nat Commun.

[B51] Lebeau A, Bruyere D, Roncarati P, Peixoto P, Hervouet E, Cobraiville G, Taminiau B, Masson M, Gallego C, Mazzucchelli G (2022). HPV infection alters vaginal microbiome through down-regulating
host mucosal innate peptides used by Lactobacilli as amino acid
sources. Nat Commun.

[B52] Lehmann BD, Bauer JA, Chen X, Sanders ME, Chakravarthy AB, Shyr Y, Pietenpol JA (2011). Identification of human triple-negative breast cancer subtypes
and preclinical models for selection of targeted therapies. J Clin Invest.

[B53] Li N, Ma WT, Pang M, Fan QL, Hua JL (2019). The commensal microbiota and viral infection: A comprehensive
review. Front Immunol.

[B54] Li R, Li J, Zhou X (2024). Lung microbiome: New insights into the pathogenesis of
respiratory diseases. Signal Transduct Target Ther.

[B55] Li X, Xiang F, Liu T, Chen Z, Zhang M, Li J, Kang X, Wu R (2024). Leveraging existing 16S rRNA gene surveys to decipher microbial
signatures and dysbiosis in cervical carcinogenesis. Sci Rep.

[B56] Liu NN, Yi CX, Wei LQ, Zhou JA, Jiang T, Hu CC, Wang L, Wang YY, Zou Y, Zhao YK (2023). The intratumor mycobiome promotes lung cancer progression via
myeloid-derived suppressor cells. Cancer Cell.

[B57] Liu Y, Zhao X, Wu F, Chen J, Luo J, Wu C, Chen T (2024). fectiveness of vaginal probiotics Lactobacillus crispatus chen-01
in women with high-risk HPV infection: A prospective controlled pilot
study. Aging (Albany NY).

[B58] Louis P, Hold GL, Flint HJ (2014). The gut microbiota, bacterial metabolites, and colorectal
cancer. Nat Rev Microbiol.

[B59] Luo YC, Huang XT, Wang R, Lin YJ, Sun JX, Li KF, Wang DY, Yan Y, Qiao YK (2025). Advancements in understanding tumor-resident bacteria and their
application in cancer therapy. Mil Med Res.

[B60] Mima K, Nishihara R, Qian ZR, Cao Y, Sukawa Y, Nowak JA, Yang J, Dou R, Masugi Y, Song M (2016). Fusobacterium nucleatum in colorectal carcinoma tissue and
patient prognosis. Gut.

[B61] Moscicki AB, Shiboski S, Broering J, Powell K, Clayton L, Jay N, Darragh TM, Brescia R, Kanowitz S, Miller SB (1998). The natural history of human papillomavirus infection as measured
by repeated DNA testing in adolescent and young women. J Pediatr.

[B62] Nejman D, Livyatan I, Fuks G, Gavert N, Zwang Y, Geller LT, Rotter-Maskowitz A, Weiser R, Mallel G, Gigi E (2020). The human tumor microbiome is composed of tumor type-specific
intracellular bacteria. Science.

[B63] Norenhag J, Du J, Olovsson M, Verstraelen H, Engstrand L, Brusselaers N (2020). The vaginal microbiota, human papillomavirus and cervical
dysplasia: A systematic review and network meta-analysis. BJOG.

[B64] Nougayrède JP, Homburg S, Taieb F, Boury M, Brzuszkiewicz E, Gottschalk G, Buchrieser C, Hacker J, Dobrindt U, Oswald E (2006). Escherichia coli induces DNA double-strand breaks in eukaryotic
cells. Science.

[B65] Öz HH, Zhou B, Voss P, Carevic M, Schroth C, Frey N, Rieber N, Hector A, Hartl D (2016). Pseudomonas aeruginosa airway infection recruits and modulates
neutrophilic myeloid-derived suppressor cells. Front Cell Infect Microbiol.

[B66] Parida S, Wu S, Siddharth S, Wang G, Muniraj N, Nagalingam A, Hum C, Mistriotis P, Hao H, Talbot CC (2021). A procarcinogenic colon microbe promotes breast tumorigenesis and
metastatic progression and concomitantly activates notch and β-Catenin
axes. Cancer Discov.

[B67] Pathak JL, Yan Y, Zhang Q, Wang L, Ge L (2021). The role of oral microbiome in respiratory health and
diseases. Respir Med.

[B68] Pei B, Peng S, Huang C, Zhou F (2024). Bifidobacterium modulation of tumor immunotherapy and its
mechanism. Cancer Immunol Immunother.

[B69] Peng Y, Gu J, Liu F, Wang P, Wang X, Si C, Gong J, Zhou H, Qin A, Song F (2024). Integrated analysis of microbiota and gut microbial metabolites
in blood for breast cancer. mSystems.

[B70] Perou CM, Sørlie T, Eisen MB, van de Rijn M, Jeffrey SS, Rees CA, Pollack JR, Ross DT, Johnsen H, Akslen LA (2000). Molecular portraits of human breast tumours. Nature.

[B71] Pleguezuelos-Manzano C, Puschhof J, Rosendahl Huber A, van Hoeck A, Wood HM, Nomburg J, Gurjao C, Manders F, Dalmasso G, Stege PB (2020). Mutational signature in colorectal cancer caused by genotoxic
pks+ Escherichia coli. Nature.

[B72] Ravel J, Gajer P, Abdo Z, Schneider GM, Koenig SS, McCulle SL, Karlebach S, Gorle R, Russell J, Tacket CO (2011). Vaginal microbiome of reproductive-age women. Proc Natl Acad Sci U S A.

[B73] Recharla N, Geesala R, Shi XZ (2023). Gut microbial metabolite butyrate and its therapeutic role in
inflammatory bowel disease: A literature review. Nutrients.

[B74] Ridlon JM, Kang DJ, Hylemon PB, Bajaj JS (2014). Bile acids and the gut microbiome. Curr Opin Gastroenterol.

[B75] Rothschild D, Weissbrod O, Barkan E, Kurilshikov A, Korem T, Zeevi D, Costea PI, Godneva A, Kalka IN, Bar N (2018). Environment dominates over host genetics in shaping human gut
microbiota. Nature.

[B76] Routy B, Le Chatelier E, Derosa L, Duong CPM, Alou MT, Daillère R, Fluckiger A, Messaoudene M, Rauber C, Roberti MP (2018). Gut microbiome influences efficacy of PD-1-based immunotherapy
against epithelial tumors. Science.

[B77] Routy B, Lenehan JG, Miller WH, Jamal R, Messaoudene M, Daisley BA, Hes C, Al KF, Martinez-Gili L, Punčochář M (2023). Fecal microbiota transplantation plus anti-PD-1 immunotherapy in
advanced melanoma: A phase I trial. Nat Med.

[B78] Rubinstein MR, Wang X, Liu W, Hao Y, Cai G, Han YW (2013). Fusobacterium nucleatum promotes colorectal carcinogenesis by
modulating E-cadherin/β-catenin signaling via its FadA
adhesin. Cell Host Microbe.

[B79] Sánchez-Alcoholado L, Ramos-Molina B, Otero A, Laborda-Illanes A, Ordóñez R, Medina JA, Gómez-Millán J, Queipo-Ortuño MI (2020). The role of the gut microbiome in colorectal cancer development
and therapy response. Cancers (Basel).

[B80] Sfanos KS, De Marzo AM (2012). Prostate cancer and inflammation: The evidence. Histopathology.

[B81] Sfanos KS, Yegnasubramanian S, Nelson WG, De Marzo AM (2018). The inflammatory microenvironment and microbiome in prostate
cancer development. Nat Rev Urol.

[B82] Song M, Chan AT, Sun J (2020). Influence of the gut microbiome, diet, and environment on risk of
colorectal cancer. Gastroenterology.

[B83] Sørlie T, Perou CM, Tibshirani R, Aas T, Geisler S, Johnsen H, Hastie T, Eisen MB, van de Rijn M, Jeffrey SS (2001). Gene expression patterns of breast carcinomas distinguish tumor
subclasses with clinical implications. Proc Natl Acad Sci U S A.

[B84] Sun Y, Wen M, Liu Y, Wang Y, Jing P, Gu Z, Jiang T, Wang W (2023). The human microbiome: A promising target for lung cancer
treatment. Front Immunol.

[B85] Sung H, Ferlay J, Siegel RL, Laversanne M, Soerjomataram I, Jemal A, Bray F (2021). Global cancer statistics 2020: GLOBOCAN estimates of incidence
and mortality worldwide for 36 cancers in 185 countries. CA Cancer J Clin.

[B86] Tamarelle J, Thiébaut ACM, de Barbeyrac B, Bébéar C, Ravel J, Delarocque-Astagneau E (2019). The vaginal microbiota and its association with human
papillomavirus, Chlamydia trachomatis, Neisseria gonorrhoeae and Mycoplasma
genitalium infections: A systematic review and meta-analysis. Clin Microbiol Infect.

[B87] Thomas AM, Manghi P, Asnicar F, Pasolli E, Armanini F, Zolfo M, Beghini F, Manara S, Karcher N, Pozzi C (2019). Metagenomic analysis of colorectal cancer datasets identifies
cross-cohort microbial diagnostic signatures and a link with choline
degradation. Nat Med.

[B88] Tong S, Huang K, Xing W, Chu Y, Nie C, Ji L, Wang W, Tian G, Wang B, Yang J (2024). Unveiling the distinctive variations in multi-omics triggered by
TP53 mutation in lung cancer subtypes: An insight from interaction among
intratumoral microbiota, tumor microenvironment, and
pathology. Comput Biol Chem.

[B89] Urbaniak C, Gloor GB, Brackstone M, Scott L, Tangney M, Reid G (2016). The microbiota of breast tissue and its association with breast
cancer. Appl Environ Microbiol.

[B90] Wang H, Ma Y, Li R, Chen X, Wan L, Zhao W (2019). Associations of cervicovaginal lactobacilli with high-risk human
Papillomavirus infection, cervical intraepithelial neoplasia, and cancer: A
systematic review and meta-analysis. J Infect Dis.

[B91] Wen Q, Wang S, Min Y, Liu X, Fang J, Lang J, Chen M (2025). Associations of the gut, cervical, and vaginal microbiota with
cervical cancer: a systematic review and meta-analysis. BMC Womens Health.

[B92] Whipps J, Lewis K, Cooke R, Burge M (1988). Mycoparasitism and plant disease control.

[B93] Wu S, Rhee KJ, Albesiano E, Rabizadeh S, Wu X, Yen HR, Huso DL, Brancati FL, Wick E, McAllister F (2009). A human colonic commensal promotes colon tumorigenesis via
activation of T helper type 17 T cell responses. Nat Med.

[B94] Xu W, Li Y, Liu L, Xie J, Hu Z, Kuang S, Fu X, Li B, Sun T, Zhu C (2024). Icaritin-curcumol activates CD8+ T cells through regulation of
gut microbiota and the DNMT1/IGFBP2 axis to suppress the development of
prostate cancer. J Exp Clin Cancer Res.

[B95] Yan C, Chen Y, Tian Y, Hu S, Wang H, Zhang X, Chu Q, Huang S, Sun W (2025). The emerging role of microbiota in lung cancer: A new perspective
on lung cancer development and treatment. Cell Oncol (Dordr).

[B96] Yu T, Guo F, Yu Y, Sun T, Ma D, Han J, Qian Y, Kryczek I, Sun D, Nagarsheth N (2017). Fusobacterium nucleatum promotes chemoresistance to colorectal
cancer by modulating autophagy. Cell.

[B97] Zhang D, Li S, Wang N, Tan HY, Zhang Z, Feng Y (2020). The cross-talk between gut microbiota and lungs in common lung
diseases. Front Microbiol.

[B98] Zhang W, Ge Y, Yao L, Yan Q, Wei J, Yin Y, Liu B (2025). Changes of microbiome in Human Papillomavirus infection and
cervical cancer: A systematic review and meta-analysis. Cancer Rep (Hoboken).

[B99] Zhang X, Wang C, Shan S, Liu X, Jiang Z, Ren T (2016). TLR4/ROS/miRNA-21 pathway underlies lipopolysaccharide instructed
primary tumor outgrowth in lung cancer patients. Oncotarget.

[B100] Zhong W, Wu K, Long Z, Zhou X, Zhong C, Wang S, Lai H, Guo Y, Lv D, Lu J, Mao X (2022). Gut dysbiosis promotes prostate cancer progression and docetaxel
resistance via activating NF-κB-IL6-STAT3 axis. Microbiome.

[B101] Zhao Y, Liu Y, Li S, Peng Z, Liu X, Chen J, Zheng X (2021). Role of lung and gut microbiota on lung cancer
pathogenesis. J Cancer Res Clin Oncol.

